# Identifying and prioritizing educational content from a malpractice claims database for clinical reasoning education in the vocational training of general practitioners

**DOI:** 10.1007/s10459-022-10194-8

**Published:** 2022-12-19

**Authors:** Charlotte G. M. van Sassen, Pieter J. van den Berg, Silvia Mamede, Lilian Knol, Manon P. Eikens-Jansen, Walter W. van den Broek, Patrick J. E. Bindels, Laura Zwaan

**Affiliations:** 1 Department of General Practice, Erasmus Medical Center Rotterdam, Rotterdam, The Netherlands; 2 Institute of Medical Education Research Rotterdam (iMERR), Erasmus Medical Center Rotterdam, Rotterdam, The Netherlands; 3 Department of Psychology, Education and Child Studies, Erasmus School of Social and Behavioral Sciences, Rotterdam, The Netherlands; 4 VvAA, Orteliuslaan 750, 3528 BB Utrecht, The Netherlands

**Keywords:** Clinical case vignette, Clinical reasoning education, Diagnostic errors, General practice, Liability insurer, Malpractice claim, Medical education, Primary care, Vocational training, Diagnostic errors, Medical education, Family practice, General practice, Malpractice, Medical errors, Missed diagnosis, Primary care, Professional misconduct, Patient safety

## Abstract

Diagnostic reasoning is an important topic in General Practitioners’ (GPs) vocational training. Interestingly, research has paid little attention to the content of the cases used in clinical reasoning education. Malpractice claims of diagnostic errors represent cases that impact patients and that reflect potential knowledge gaps and contextual factors. With this study, we aimed to identify and prioritize educational content from a malpractice claims database in order to improve clinical reasoning education in GP training. With input from various experts in clinical reasoning and diagnostic error, we defined five priority criteria that reflect educational relevance. Fifty unique medical conditions from a malpractice claims database were scored on those priority criteria by stakeholders in clinical reasoning education in 2021. Subsequently, we calculated the mean total priority score for each condition. Mean total priority score (min 5–max 25) for all fifty diagnoses was 17,11 with a range from 13,89 to 19,61. We identified and described the fifteen highest scoring diseases (with priority scores ranging from 18,17 to 19,61). The prioritized conditions involved complex common (e.g., cardiovascular diseases, renal insufficiency and cancer), complex rare (e.g., endocarditis, ectopic pregnancy, testicular torsion) and more straightforward common conditions (e.g., tendon rupture/injury, eye infection). The claim cases often demonstrated atypical presentations or complex contextual factors. Including those malpractice cases in GP vocational training could enrich the illness scripts of diseases that are at high risk of errors, which may reduce diagnostic error and related patient harm.

## Introduction

Diagnostic errors are worldwide the most common, costly and severe error type in malpractice claim (Saber Tehrani et al., 2013). They can lead to severe consequences for patients, with studies showing moderate to severe damage up to 86,8% (Singh et al., 2013) and they more often contribute to patient death than other types of errors (Zwaan et al., 2010). Recent estimates reveal that approximately 5% of all patients presenting to primary care are subject to diagnostic error (Singh et al., 2014). Improving clinical reasoning education has been identified as an important way to reduce diagnostic error (Graber et al., 2018; National Academies of Sciences Engineering & Medicine, 2015). The need for a structured, longitudinal implementation of clinical reasoning education in medical schools in all specialties has been widely advocated in literature recently (Cooper et al., 2021; Kononowicz et al., 2020), as existing training programs may not provide adequate education related to diagnostic safety (Graber et al., 2018)**.** Ideally, clinical reasoning education should be introduced in undergraduate (preclinical) medicine, starting with low-complex cases with typical presentations for developing basic medical knowledge. Throughout medical school and further into residency it should advance with more complex, rare or atypical case presentations that require a deeper level of reasoning and understanding, adapted to the level of expertise of the learner (Cooper et al., 2021; Leppink & Duvivier, 2016; Schmidt and Mamede 2015; Van Merriënboer & Sweller, 2010). This way, illness scripts (i.e., mental representations of the diseases) will be enriched over time, allowing for the learners to recognize both typical and atypical disease presentations, which contributes to better performance in diagnostic reasoning (Charlin et al., 2007; Eva et al., 1998; Lubarsky et al., 2015; Schmidt et al., 1990). Although all studies emphasize the importance of a careful choice of content for the case vignettes, there has been little attention in research on how to determine the most appropriate content of the cases from which students learn in clinical reasoning education. This is surprising, since there is increasing evidence that the content of the case determines what the students learn (Sherbino & Norman, 2014). Ideally, case content should reflect information that trainees need to know but have not yet mastered because of insufficient exposure (Norman, 2012). These knowledge gaps could entail various aspects of a clinical case, such as medical-technical content (atypical presentations, rare diseases), awareness of the risk of under- or overdiagnosis or the impact of the patient harm that may result from missing or delaying the diagnosis. In addition to these case-specific factors, there are many other contextual factors that influence diagnostic decision making, e.g. patient perspectives, the physician–patient relationship, the availability of diagnostic tests or support staff and the system or environment where care is rendered (Durning et al., 2011, 2020; Weiner & Schwartz, 2016). However, both case-specific—and contextual factors are not always reflected in the fictive, clinical case vignettes that are most frequently used in current clinical reasoning education. Therefore, it would be valuable to expand clinical reasoning education by using a larger variety of sources. Malpractice claims for example, reflect knowledge gaps as well as real-life contextual factors of a clinical case. In addition, those cases also reflect situations that impacted patients. By learning from malpractice claim cases, advanced students (i.e. residents) may learn from the mistakes of their peers (Fischer et al., 2006), thereby deriving educational benefit from diagnostic errors (Eva, 2009). However, not every claim on diagnostic error is suitable for clinical reasoning education. For example, diagnoses that are so rare that it is unlikely a doctor will encounter them during their career, or diagnoses that have a high risk of overdiagnosis, may be less suitable for more extensive clinical reasoning education. The aim of this study was to identify and prioritize, using priority criteria, conditions with the highest expected educational value for GP residents from our national malpractice claims database on diagnostic error for clinical reasoning education. This could be a way to complement the current clinical reasoning curriculum.

## Methods

### Study design

This study consisted of two parts. In the first part, experts in clinical reasoning education and diagnostic error defined five criteria that were considered important in determining the educational relevance of a medical condition. In the second part, three types of stakeholders in clinical reasoning education participated in ranking fifty unique missed diagnoses from a claims database on their educational relevance, using the five pre-defined priority criteria from the first part. Based on the scores, a top fifteen of the medical conditions with the highest educational value for clinical reasoning education was determined.

### Setting

The current clinical reasoning curriculum of our post-graduate, three-year GP vocational training at Erasmus MC Rotterdam consists of eight themes that are distributed over all three years of training. Clinical reasoning education is embedded in learning sessions during regular weekly one-day educational sessions supervised by our teaching staff or during daily clinical practice supervised one-on-one by senior GPs. Each theme includes various fictive case vignettes on different diagnoses for trainees to solve. Case content varies from typical presentations to atypical, but generally leans more towards typical and common disease presentations.

### Participants

#### Part 1: setting criteria for educational priority

For the first part of the study, we created a local body of experts to formulate educational priority criteria, existing of five senior GPs from our teaching staff involved in clinical reasoning education and one expert in the field of diagnostic error from the department of General Practice at Erasmus MC. Moreover, we asked four international experts on clinical reasoning education and diagnostic error as well as one representative of the liability insurance company and one representative of the Dutch Patient Federation for feedback on these criteria. The selection of the international experts from our network was based on their expertise in clinical reasoning education and/or diagnostic error.

#### Part 2 ranking the claims database with educational priority criteria

For the second part of the study, we included three types of stakeholders of medical education. Firstly, we included twenty-five third-year GPs in training. Being nearly graduated as a GP, residents have an important understanding of their own needs, knowledge and the current curriculum. Secondly, twenty-five daily supervisors of these trainees (senior GPs who supervise the residents in clinical practice in a one-on-one situation), who have a good impression of the practical knowledge gaps encountered by the trainees in daily practice were included. And lastly, nineteen lecturers of GP vocational training, who teach and/or supervise trainees during their weekly scientific sessions and have a good overview of the content of the current medical curriculum and the needs of the trainees were included.

### Materials and procedure

#### Part 1: setting criteria for educational priority

Priority criteria and the methodology to score them do exist for research questions but not yet for educational content (Rudan et al., 2008). Based on the Child Health and Nutrition Research Initiative (CHNRI) methodology for assessing research questions, the local body of experts adapted the existing research criteria to five criteria for educational relevance during an in-person panel discussion, also considering the framework for measurement of diagnostic safety (Olson et al., 2018), to identify conditions prone to diagnostic error. Subsequently, independent feedback on these criteria was solicited from the international experts on clinical reasoning education and diagnostic error, a representative of the liability insurance company and a representative of the Dutch Patient Federation to refine the criteria. See Table 1 for the criteria.

**Table 1:** Criteria for setting priorities in clinical reasoning education content as formulated by a body of experts in clinical reasoning education

1	Suitability: the disease is suitable for developing a clinical case vignette for clinical reasoning education
	Is the complexity of the disease and the content of the claim suitable for Clinical Reasoning Education? This means that the disease can present in different ways or atypically and sufficient clinical reasoning is needed for diagnosing it. The disease should be well defined and it should not be a diagnosis per exclusionem
2	Incidence of the disease: the disease is common in general practice
3	Patient-related harm reduction: teaching about the disease has sufficient potential to reduce diagnostic error and diagnosis-related harm for the patient
	Are the consequences of a diagnostic error for this disease high enough for the patient? For example, a missed common cold has relatively low impact on the patient
4	Knowledge gap: the GP in training has too little knowledge about this disease
	For example the disease is insufficiently covered in current education/training or in the existing protocols or guidelines
5	Overdiagnosis, overtesting or overtreatment: By teaching the disease there will be risk of harmful overdiagnosis, overtesting or overtreatment^1^

Criteria to be scored on a five-point scale for each diagnosis, where 1 is ‘not at all’ and 5 is ‘very much’. Total priority scores range from 5 to 25 points per disease. For some criteria an extra description was added to clarify the criterion

^1^Criterion 5 is formulated paradoxically and the score needs to be reversed before calculating the total priority score for a disease

#### Part 2: ranking the claims database with educational priority criteria

#### Claims database

For this study, the liability insurance company covering 85% of the GP practices in the Netherlands made their claim database with cases filed between 2012 and 2017 available to us for educational purposes. The claim information was entered into a database and summarized by the insurance company, but the insurance company was not further involved in the analysis and interpretation of the study findings. The most important variables in the dataset were a description of the claim, a short summary of the response of the medical advisor, the liability outcome and the amount of indemnity paid (see Table 3 for a description of all available variables in the anonymous abstract of the claims database). We focused on the legitimate complaints (as judged by the insurance company) and on diagnostic error only. Between 2012 and 2017, eight hundred thirty-five (835) claims related to a diagnostic error against GPs were documented and closed. One hundred thirty-eight (138, 16.5%) of them were accepted as legitimate complaints for which indemnity was paid for a total amount of €4.7 million. Since it was not always immediately clear from the data which specific diagnosis was missed in a claim, two GPs (one study investigator and one independent lecturer from the department of GP) independently identified the specific missed diagnosis in each of the claims, using the description of the claim and the short summary of the medical advisor. When missed diagnoses did not become directly clear from the description or summary, or when there was no consensus between the GPs, the liability insurance company was asked to check the full record for the specific missed diagnosis. Disagreements were resolved by the GPs by discussion. Subsequently, the missed diagnoses were first ranked based on frequency of occurrence in the claims database and second on average amount of indemnity paid. This produced a list of a total of fifty (50) unique missed diagnoses (see Table 4 for the full list).

#### Ranking

All stakeholders received a link to an online Qualtrics questionnaire, which is a web-based survey tool. In the Qualtrics questionnaire, after giving informed consent, the participants were asked to score the educational relevance of the diagnoses of the claims database with the five priority criteria (see Table 1) on a five-point scale. Each diagnosis could be rated with 1 to 5 points for each criterion, where 1 is ‘not at all’ and 5 is ‘very much’. Instructions regarding the use of the criteria for scoring educational priority were given beforehand. Since scoring fifty diagnoses would take too much time per participant, each participant was randomly assigned to scoring 25 of the 50 diagnoses (i.e. either the odd or even numbers). For each diagnosis, we gave a short description of (a typical example of) the missed diagnosis from the claims database to clarify the main cause of the missed diagnosis (see Table 5 for examples).

### Analysis

#### Part 2: ranking the claims database with educational priority criteria

Total priority score for each diagnosis could range from minimum 5 to maximum 25 points. The higher the score, the more relevant the disease was considered to be for education. Since formulation of criterion number five was paradoxical compared to the other four criteria (negatively formulated instead of positively), we reversed the score of this criterion first. We then calculated the mean score for each of the five criteria and the mean total priority score for each diagnosis using SPSS Statistics version 25 for Windows (IBM). We obtained a ranking of most relevant diseases for clinical reasoning education, of which we will present the top fifteen in the results section.

## Results

### Part 2: ranking the claims database with educational priority criteria

### Participants

15 out of 25 invited (60%) GPs in training, 10 out of 25 invited (40%) supervisors of GPs in training and 12 out of 19 invited (63%) lecturers/teachers of the GP vocational training completed the questionnaire**.** In total, the ranking was based on 37 participants, 19 participants (51.4%) scored the odd cases and 18 (48.6%) the even cases.

### Ranking list of most relevant diagnoses for education

In Table 2, the fifteen conditions that received the highest educational priority scores are presented (see Table 6 for the complete prioritized list). In the first column the original position of the diagnosis (based on frequency of occurrence and average amount of indemnity paid in the claims database) is presented, which shows that also diagnoses that had originally lower positions in the ranked claims database were considered educational priorities after the prioritization exercise. The overall mean total priority score for all fifty diagnoses was 17,11 (SD 1,31) and ranged from 13,89 to 19,61. The priority score of the top-fifteen missed diagnoses ranged from 18,17 to 19,61.

**Table 2:** Top-fifteen conditions from claim database that received the highest score on educational priority criteria from various stakeholders in medical education (e.g., trainees, supervisors, teachers of GP vocational training). Results include mean total priority scores based on the total of five priority criteria and mean scores per criterion

Original position in malpractice claim database (based on frequency and indemnity paid)	Prioritized missed diagnoses	Mean total priority score (SD)	Mean score suitability/complexity	Mean score incidence of the disease	Mean score patient-related harm	Mean score knowledge gap	Mean score overdiagnosis, -testing or –treatment (reversed; high scores represent low risk)	Number of respondents
	Mean (SD) [range] of all 50 diagnoses	17,11 (1,31) [13,89–19,61]	3,85 (0,58) [2,17–4,83]	2,85 (0,88) [1,53–4,53]	3,87 (0,56) [2,50–4,79]	3,10 (0,48) [1,83–4,22]	3,44 (0,36) [2,68–4,11]	
6	1 Cerebrovascular accident	19,61 (2,30)	4,56	4,39	4,06	3,00	3,61	18
45	2 Cardiopulmonary instability	19,42 (2,80)	4,63	3,47	4,79	2,95	3,58	19
14	3 Eye infection	18,67 (2,72)	4,06	3,44	4,17	3,33	3,67	18
2	4 Myocardial infarction	18,61 (2,59)	4,83	3,94	4,00	2,67	3,17	18
18	5 Ectopic pregnancy	18,61 (1,72)	4,50	2,56	4,50	3,00	4,06	18
9	6 Arterial occlusion lower leg	18,58 (1,77)	4,53	2,37	4,53	3,32	3,84	19
8	7 Chronic coronary sclerosis	18,56 (3,05)	4,50	3,89	4,06	3,22	2,89	18
40	8 Sinus thrombosis	18,50 (2,36)	4,67	2,06	4,44	3,72	3,61	18
5	9 Tendon rupture	18,47 (2,25)	3,89	3,37	4,11	3,63	3,47	19
3	10 Cancer	18,47 (2,29)	4,21	3,84	4,21	2,95	3,26	19
13	11 Deep venous thrombosis	18,32 (2,26)	4,16	3,74	4,16	2,84	3,42	19
31	12 Renal insufficiency	18,32 (2,54)	4,26	3,58	4,11	3,05	3,32	19
4	13 Testicular torsion	18,28 (1,96)	4,28	2,33	4,39	3,33	3,94	18
7	14 Tendon injury	18,26 (2,23)	3,84	2,95	4,26	3,26	3,95	19
22	15 Endocarditis	18,17 (2,60)	4,50	1,72	4,39	3,89	3,67	18

## Discussion

The aim of this study was to identify and prioritize relevant educational content from a claims database of diagnostic errors in order to enrich clinical reasoning education in GP training. In this article, we presented the fifteen conditions from a Dutch claims database that received the highest score on newly formulated educational priority criteria from various stakeholders in medical education (i.e., trainees, supervisors, teachers) of the department of general practice at Erasmus MC, Rotterdam, The Netherlands.

Looking closely at the top-fifteen conditions, the conditions could be categorized in different groups with similar characteristics, for example complex common conditions, complex rare conditions and straightforward common conditions. Common but complex conditions are represented in the prioritized list by cardiovascular diseases (cerebrovascular accident, cardiopulmonary instability, myocardial infarction, arterial occlusion, coronary sclerosis and deep venous thrombosis), renal insufficiency and cancer. These conditions recur regularly in international malpractice claims lists. Although lower-priority conditions often involve diagnostic errors due to late referral or failure to order the appropriate diagnostic test, these conditions more often involve atypical disease presentations or complex contextual factors that allow alternative diagnoses to explain symptoms. For instance, a cerebrovascular accident was confused with alcohol intoxication in a known alcoholic, a myocardial infarction was mistaken for an exacerbation of COPD, or an arterial occlusion in a leg was preceded by trauma that provided a good explanation for the pain.

Another example of the need for a greater diversity of atypical case histories in education is the rarer diagnosis testicular torsion. While testicular torsion is covered in the curriculum, it ranks thirteenth in the educational priority list and a notable fourth place in the claims database is occupied by this condition. Although this is in line with findings in international literature (Colaco et al., 2015; Najaf-Zadeh et al., 2011; Osman & Collins, 2011; Raine, 2011; Ryan et al., 2020), it is remarkable since the incidence of testicular torsion in general practice is low (around 1:1800–4000 per year in children/adolescents till 25 years of age (Zhao et al., 2011)) and the differential diagnosis of testicular complaints is not very extensive. Moreover, the diagnostic examination for excluding a torsion (ultrasonography) is relatively easy to perform and not invasive. For these reasons one would not expect so many misdiagnoses of testicular torsion in the claim database and its subsequent high ranking on the educational priority list. Again for this disease, the descriptions of the claim cases commonly involve an atypical or complex presentation, e.g., non-acute abdominal pain (Pogorelić et al., 2013) or vomiting rather than acute severe pain in the testicle, as is currently usually taught to our students.

Other rare conditions that emerge from the priority list as urgent topics for clinical reasoning education are diseases that are complex by nature, to which physicians are insufficiently exposed in daily practice due to their low incidence, for example the diagnoses endocarditis, sinus thrombosis and extra-uterine pregnancy. More extensive exposure to these diseases during clinical reasoning education can facilitate their recognition and reduce diagnostic errors.

Not only complex or rare diseases, but also straightforward and common conditions appear on the shortlist, such as a tendon rupture/injury or eye infection. Here, physicians might be insufficiently aware of the risk and consequences of misdiagnosis for these relatively easy and common diagnoses. In a Swedish report on diagnostic errors, tendon rupture ranks fifth, after cancer, fractures, infections and heart disease in primary care and even second in the emergency department (Fernholm et al., 2019). For most GPs, the consequences of missing a tendon rupture are not as clear as for missing a myocardial infarction or cerebrovascular accident. Hence, more exposure to these conditions to create more awareness of what can go wrong, where the risks of misdiagnosis lie and what the possible serious consequences are, are considered to have added value for clinical reasoning education.

Besides the need for more attention to rare diseases and awareness of the consequences of missing a diagnosis, this priority list particularly emphasizes the need to include more atypical presentations or cases with complex contextual factors in the clinical reasoning curriculum for general practitioners in training so that experience can be gained with a wider range of examples. This may enrich the illness scripts (i.e., the mental representations of diseases) in physicians’ minds, which has proven to be an effective learning strategy in clinical reasoning education, especially for more advanced learners (Charlin et al., 2007; Eva et al., 1998; Lubarsky et al., 2015; Schmidt et al., 1990). In combination with other proven effective educational strategies to improve diagnostic performance such as deliberate reflection (Mamede et al., 2012, 2014; Prakash et al., 2019) and self-explanation (Chamberland et al., 2011, 2015), malpractice claim cases might provide a unique opportunity to optimize educational content, thereby potentially reducing diagnostic error and related patient-harm.

### Strengths and limitations

As far as we know, this is the first study that systematically prioritized educational content from a malpractice claim database using newly formulated educational priority criteria. The claims database identifies specific conditions that are prone to diagnostic errors, and by definition have impact on patients. These prioritized diseases could be used to help selecting conditions to complement the clinical reasoning curriculum. In addition to this, the database presents real-life, practical examples of atypical presentations of a disease or cases with complex contextual factors, such as doctor-patient relationship and communication, patient perspectives and the system or environment where care is rendered. Practice with these cases could be especially beneficial for more experienced, post-graduate learners. However, adding too many conditions with atypical disease presentations could lead to more diagnostic testing to rule out those atypical presentations and therefore to (possible harmful) overtesting and overdiagnosis. It is therefore important to balance the use of malpractice claim cases in the curriculum with cases reflecting more typical and common disease presentations. This supports the argument that exposure to atypical disease presentations is more appropriate for advanced students, as for them it complements their experience in clinical practice where they see the more common and typical disease presentations.

Although the validity of the criteria that we formulated for this study was not extensively tested, we based them on the existing criteria used by CHNRI and Olson’s framework for measurement of diagnostic safety (Olson et al., 2018; Rudan et al., 2008). Moreover, we consulted various local and international experts on clinical reasoning education and diagnostic error, as well as GPs, a liability insurer representative and the Patient Federation. The criteria were formulated after extensive discussion and represent relevant aspects that are important in assessing educational relevance of a condition. The majority of the criteria, such as incidence, complexity of a condition, the risk for overdiagnosis and impact of a missed diagnosis on the patient are independent of location or educational curricula and therefore widely applicable. The participants that scored the diagnoses in this study represent the most important stakeholders of medical education of GP training, albeit all from one university centre. In other stages of medical education, other specialties and other university centres, educational curricula and consequent scoring of knowledge gaps might differ and therefore conditions might be prioritized differently. Developers of medical education could use the priority criteria to filter out the most relevant conditions for their setting, either provided by our claim list or from their local claims databases.

Despite our Dutch malpractice claim database not being a very large dataset in absolute numbers, it does reflect the majority (85%) of all claims filed against GPs in our country during a five-year-period. Moreover, the diseases recorded in our database do appear to be consistent with those of other larger claims databases of high-income countries worldwide (Fernholm et al., 2019; Wallace et al., 2013). Low- or middle-income countries may have different conditions represented in their local claim databases, for example due to different incidences of diseases or less availability of staff and diagnostic tests. However, atypical case presentations remain comparable worldwide, independent of time, setting and place, as it deals primarily with medical technical content and less with system- and management factors. Therefore, we expect that the diseases recurring in our priority list can be largely generalized to other (high-income) countries, and low- and middle-income countries could also use their local (claims) databases to define their own educational priorities with the formulated priority criteria.

### Conclusion and recommendations

With the methods and results from this unique study, we can make specific suggestions to the developers of medical education on how to obtain a shortlist of conditions from (their local) malpractice claims that have high educational relevance because they are prone to error. It would be recommendable that educational developers get insight in the aggregated data of medical liability insurance companies and/or disciplinary boards to select relevant content for medical education. This allows not only for training with complex conditions that practitioners are insufficiently exposed to in daily practice due to their low incidence, but also for recognizing possible serious consequences of missing a relatively common and straightforward diagnosis. Additionally, for more expert learners, it might result in developing broader illness scripts for conditions that are usually basically covered in the curriculum in its typical form of presentation, but can present atypically or with complex contextual factors. This might contribute to gaining experience with a larger set of examples, which can help recognizing (atypical presentation of) these diseases in future patients, reducing the number of misdiagnoses and related patient harm. Furthermore, using non-fictive malpractice claim cases, encompassing all circumstantial and contextual factors that contribute to diagnostic error, as vignettes for clinical reasoning education might be a valuable method for further optimizing educational content, but this requires more research on its effectiveness.

## Appendix

See Tables 3 , 4 , 5  and 6.

**Table 3:** Variables in the anonymous abstract of the claims database between 2012–2017

Short description of the claim
Short summary of the response of the medical advisor of the liability insurer
Amount of indemnity paid
Time between the incident and reporting of the case
Time between reporting and closing of the case
Type of employee involved (GP, GP in training, GP assistant)
*In what part of the care process the incident took place*
Patient history and clinical examination
Coordination of care
Interpretation of diagnostic tests
Ordering of diagnostic tests
Follow-up
*Liability outcome*
Accepted
Denied
Culpable w/o damages
Settlement
Closed w/o decision
Patient age
Patient sex
Treated organ
Affected organ
Consequential damage according to patient
Consequential damage according to medical expert
*Cause of the incident*
Human
Organizational

**Table 4:** List of all missed diagnoses from the claims database 2012–2017, ordered first by frequency of occurrence and second by average amount of indemnity paid per case, rounding to thousands and above €100.000 to ten thousands (“VvAA liability Insurance company database” n.d.)

	Missed diagnosis	Frequency in database	Rounded (average) cost of indemnity per case
1	Fracture	17	€10.000
2	Myocardial infarction	14	€71.000
3	Cancer	12	€59.000
4	Testicular torsion	9	€8.000
5	Tendon rupture	8	€13.000
6	Cerebrovascular accident	5	€11.000
7	Tendon Injury	5	€9.000
8	Chronic coronary sclerosis	4	€53.000
9	Arterial occlusion lower leg	4	€48.000
10	Retinal detachment	4	€12.000
11	Luxation	4	€6.000
12	Cauda equina	3	€67.000
13	Deep venous thrombosis	3	€16.000
14	Eye Infection	3	€4.000
15	Subarachnoid hemorrhage	2	€133.000
16	Meningitis	2	€63.000
17	Intracranial injury (trauma)	2	€22.000
18	Ectopic pregnancy	2	€21.000
19	Appendicitis	2	€12.000
20	Pneumonia	2	€10.000
21	Abdominal aneurysm	2	€6.000
22	Endocarditis	1	€450.000
23	Cervical radicular syndrome	1	€200.000
24	Hemochromatosis	1	€140.000
25	Spinal abscess	1	€82.000
26	Intestinal perforation due to button cell battery ingestion	1	€44.000
27	Temporal arteriitis	1	€38.000
28	Pott’s puffy tumor	1	€37.000
29	Recurrent urinary tract infection	1	€36.000
30	Pregnancy	1	€32.000
31	Renal insufficiency	1	€31.000
32	Carpal tunnel syndrome	1	€31.000
33	Benign brain tumor	1	€26.000
34	Protein S deficiency causing lung embolism	1	€26.000
35	Hashimoto hypothyroidy	1	€23.000
36	Sudden deafness	1	€22.000
37	Nervous injury after hand trauma	1	€20.000
38	Liver disease in pregnancy	1	€16.000
39	Compartment syndrome lower leg	1	€15.000
40	Sinus thrombosis	1	€13.000
41	Urosepsis	1	€8.000
42	Venous thrombosis eye	1	€6.000
43	Scoliosis	1	€6.000
44	Hip dysplasia	1	€6.000
45	Cardiopulmonary instability	1	€5.000
46	Peritonsillar abscess	1	€2.000
47	Wound infection finger	1	< €1000
48	Wound infection with corpori alieni	1	< €1000
49	Severe vaginal blood loss	1	< €1000
50	Otitis media	1	€0

**Table 5:** Short description of (the common denominator of) the missed diagnoses of the top-fifteen educational priorities from the malpractice claim database 2012–2017

2	Myocardial infarction. The context allows for an alternative diagnosis and because of this, general practitioners make an alternative diagnosis instead of cardiac complaints (e.g., muscle pain, Tietze, COPD) and do not urgently refer to the cardiologist. The consequences vary from little harm to death
3	Cancer. Most commonly missed is breast cancer. Most claims arise because results are not passed on correctly or because advice from, for example, the radiologist is not followed. To a lesser extent, additional tests such as mammography or colonoscopy are not requested quickly enough
4	Testicular torsion. Usually there is an atypical presentation: no acute onset with severe pain in the testicle, but a longer run-in with abdominal pain or vomiting or a milder pain in the testicle that has existed for several days. Torsion is then either not thought of and thus no ultrasound is made, or antibiotics are given under the suspicion of epididymitis without making an ultrasound. The consequence is loss of a testicle/fertility problems
5	Tendon rupture. In almost all cases the diagnosis is muscle tear rather than tendon rupture. The patients have persistent symptoms and return to the clinic several times but an ultrasound scan is made only after a long time. Therefore, reconstruction of the tendon is often not possible. There is also a patient delay due to insufficient instructions on when to return
6	Cerebrovascular accident. The context provides room for an alternative diagnosis and as a result, general practitioners make an alternative diagnosis instead of CVA (e.g. alcohol abuse, carpal tunnel syndrome) and do not refer to the neurologist urgently. Consequence: permanent neurological damage, but it is not entirely clear whether this would have been preventable
7	Tendon injury. This refers to tendon injuries from (cutting) wounds. The GP forgets to test for tendon injury when assessing the wound. As a result, primary suturing of the tendon is often no longer possible, leading to loss of function
8	Chronic coronary sclerosis. The patient is not referred to the cardiologist for atypical chest pain symptoms. The guideline is not followed or the context allows for an alternative diagnosis that may explain the symptoms. The consequences vary greatly: from delay without harm to death
9	Arterial occlusion lower leg. In almost all cases, the complaints started after a trauma or arose from pre-existing morbidity of the foot, such as a nerve entrapment, edema, or thrombosis. As a result, the general practitioner explains the symptoms and does not consider the possibility of an arterial occlusion of the leg. The result is an amputation
13	Deep venous thrombosis. Claims are based on insufficient physical examination or failure to follow the guideline. Consequential damage varies from pulmonary embolism to delay of treatment without further damage
14	Infection eye (trivial, herpes). The general practitioner refers too late in the presence of alarm symptoms causing loss of vision
18	Ectopic pregnancy. Acute abdominal pain in pregnancy was not discussed by the assistant with the GP. In another case no referral to a gynecologist took place after a previous ectopic pregnancy. Consequence of this was emergency surgery, removal of fallopian tube and infertility as a result
22	Endocarditis. Patient presents with persistent general malaise and cough 8 months after aortic valve surgery. For weeks, the family physician remains on the trail of a viral respiratory infection, despite repeated presentation of patient with persistent symptoms. The result is substantial residual symptoms after cerebral and spleen infarctions
31	Renal Insufficiency. The general practitioner did not determine the kidney function for a long time while there was previously an abnormal kidney function in a patient known to have hypertension. The result is an acute kidney transplant that could and should have been performed earlier if there had been adequate renal function controls
40	Sinus thrombosis. The general practitioner failed to refer a patient with vomiting, headache and double vision timely. This caused a delay in diagnosis. The consequential loss is a longer recovery period of disability
45	Cardiopulmonary instability. The general practitioner did not sufficiently ask for the history on the telephone, so that an emergency visit was not arranged. Patient died of respiratory failure e.c.i. (dd myocardial infarction, arrhythmia or pulmonary)

**Table 6:** Complete prioritized list of claims database based on mean total priority score per diagnosis

Original position in malpractice claim database (based on frequency and indemnity paid)		Prioritized missed diagnosis	Mean total priority score	SD
6	1	Cerebrovascular accident	19,61	(2,30)
45	2	Cardiopulmonary instability	19,42	(2,80)
14	3	Eye infection	18,67	(2,72)
2	4	Myocardial infarction	18,61	(2,59)
18	5	Ectopic pregnancy	18,61	(1,72)
9	6	Arterial occlusion lower leg	18,58	(1,77)
8	7	Chronic coronary sclerosis	18,56	(3,05)
40	8	Sinus thrombosis	18,50	(2,36)
5	9	Tendon rupture	18,47	(2,25)
3	10	Cancer	18,47	(2,29)
13	11	Deep venous thrombosis	18,32	(2,26)
31	12	Renal insufficiency	18,32	(2,54)
4	13	Testicular torsion	18,28	(1,96)
7	14	Tendon injury	18,26	(2,23)
22	15	Endocarditis	18,17	(2,60)
16	16	Meningitis	18,11	(1,68)
41	17	Urosepsis	18,00	(2,98)
10	18	Retinal detachment	17,94	(2,15)
24	19	Hemochromatosis	17,94	(2,51)
36	20	Sudden deafness	17,83	(2,53)
27	21	Temporal arteriitis	17,53	(2,22)
26	22	Intestinal perforation after button cell battery ingestion	17,44	(3,07)
12	23	Cauda equina	17,39	(2,35)
32	24	Carpal tunnel syndrome	17,17	(2,41)
44	25	Hip dysplasia	17,17	(2,81)
29	26	Recurrent urinary tract infection	17,16	(1,98)
37	27	Nervous injury after hand trauma	17,05	(2,70)
1	28	Fracture	16,84	(2,46)
17	29	Intracranial injury (trauma)	16,68	(3,84)
20	30	Pneumonia	16,61	(2,48)
38	31	Liver disease in pregnancy	16,61	(2,48)
23	32	Cervical radicular syndrome	16,58	(2,73)
46	33	Tonsillar abscess	16,56	(3,13)
21	34	Abdominal aneurysm	16,37	(2,34)
43	35	Scoliosis	16,32	(3,62)
19	36	Appendicitis	16,26	(2,64)
15	37	Subarachnoid hemorrhage	16,16	(2,91)
35	38	Hashimoto hypothyroidy	16,05	(1,54)
28	39	Potts puffy tumor	16,00	(2,30)
39	40	Compartment syndrome lower leg	15,95	(2,55)
42	41	Venous thrombosis eye	15,94	(2,60)
47	42	Wound infection finger	15,89	(2,60)
25	43	Spinal abscess	15,74	(2,33)
11	44	Luxation	15,74	(2,96)
49	45	Severe vaginal blood loss	15,63	(2,54)
50	46	Otitis media	15,33	(2,14)
34	47	Protein S deficiency causing lung embolism	15,28	(2,72)
30	48	Pregnancy	14,83	(3,55)
33	49	Benign brain tumor	14,74	(2,98)
48	50	Wound infection with corpori alieni	13,89	(3,14)
